# A safety study of transumbilical single incision versus conventional laparoscopic surgery for colorectal cancer: study protocol for a randomized controlled trial

**DOI:** 10.1186/s13063-015-1067-5

**Published:** 2015-11-30

**Authors:** Yanan Wang, Ruoyan Liu, Ze Zhang, Qi Xue, Jun Yan, Jiang Yu, Hao Liu, Liying Zhao, Tingyu Mou, Haijun Deng, Guoxin Li

**Affiliations:** Department of General Surgery, Nanfang Hospital, Southern Medical University, North Guangzhou Road 1838, 510-515 Guangzhou, China; Chinese Medical Doctor Association, Beijing, China; Chinese Anti-cancer Association, Tianjin, China; Endoscopic and Laparoscopic Surgeons of Asia, Seoul, Korea; The Royal College of Surgeons of England, London, England; World Gastrointestinal and Endoscopic Doctors Association, Hongkong, China; International Association of Surgeons, Gastroenterologists and Oncologists, Kyoto, Japan; Society of American Gastrointestinal and Endoscopic Surgeons, Los Angeles, CA USA; International Gastric Cancer Association, Tokyo, Japan; Harbin Medical University Cancer Hospital, Harbin, China; The Third Affiliated Hospital of Nanchang University, Nanchang, China; Jiaozhou Central Hospital of Qingdao, Qingdao, China; The Affiliated Tumor Hospital of Zhengzhou University, Zhengzhou, China

**Keywords:** Colorectal cancer, laparoscopy, single incision, SILS, rectosigmoid, transumbilical, randomized controlled trial, study protocol

## Abstract

**Background:**

Single-incision laparoscopic surgery (SILS) is an emerging minimally invasive surgery to reduce abdominal incisions. However, despite the increasing clinical application of SILS, no evidence from large-scale, randomized controlled trials is available for assessing the feasibility, short-term safety, oncological safety, and potential benefits of SILS compared with conventional laparoscopic surgery (CLS) for colorectal cancer.

**Methods/Design:**

This is a single-center, open-label, noninferiority, randomized controlled trial. A total of 198 eligible patients will be randomly assigned to transumbilical single incision plus one port laparoscopic surgery (SILS plus one) group or to a CLS group at a 1:1 ratio. Patients ranging in age from 18 to 80 years with rectosigmoid cancer diagnosed as cT1-4aN0-2 M0 and a tumor size no larger than 5 cm are considered eligible. The primary endpoint is early morbidity, as evaluated by an independent investigator. Secondary outcomes include operative outcomes (operative time, estimated blood loss, and incision length), pathologic outcomes (tumor size, length of proximal and distal resection margins, and number of harvested lymph nodes), postoperative inflammatory and immune responses (white blood cells [WBC], neutrophil percentage [NE %], C-reactive protein [CRP], interleukin-6 [IL-6], and tumor necrosis factor-α [TNF-α]), postoperative recovery (time to first ambulation, flatus, liquid diet, soft diet, and duration of hospital stay), pain intensity, body image and cosmetic assessment, 3-year disease free survival (DFS), and 5-year overall survival (OS). Follow-up visits are scheduled for 1 and 3 months after surgery, then every 3 months for the first 2 years and every 6 months for the next 3 years.

**Discussion:**

This trial will provide valuable clinical evidence for the objective assessment of the feasibility, safety, and potential benefits of SILS plus one compared with CLS for the radical resection of rectosigmoid cancer. The hypothesis is that SILS plus one is feasible for the radical resection of rectosigmoid cancer and offers short-term safety and long-term oncological safety comparable to that of CLS, and that SILS plus one offers better cosmetic results and faster convalescence compared to CLS.

**Trial registration:**

ClinicalTrials.gov: NCT02117557 (registered on 16 April 2014).

**Electronic supplementary material:**

The online version of this article (doi:10.1186/s13063-015-1067-5) contains supplementary material, which is available to authorized users.

## Background

### Rationale

Colorectal cancer is one of the most commonly diagnosed cancers worldwide, and adequate surgical excision of the primary tumor is the only curative treatment [1, 2]. As the minimally invasive surgical era evolves and laparoscopic techniques continue to develop, conventional laparoscopic surgery (CLS) has been recommended as an alternative to open surgery for colorectal cancer. Compared with open surgery, CLS provides various advantages, including reduced pain, a shorter hospital stay, and improved cosmetic results [3–6]. Furthermore, many studies have demonstrated that its short-term safety and long-term oncological safety, in terms of DFS and OS, are comparable to those of open surgery [3–9]. These excellent results have encouraged surgeons to find more minimally invasive techniques for colorectal cancer. Recently, many of these efforts have focused on single-incision laparoscopic surgery (SILS).

SILS is performed entirely through one extraction site, typically through the umbilicus, an embryological natural orifice that can conceal a surgical scar [10]. Since it was first reported in 2008, several retrospective studies have suggested that SILS offers potential benefits over CLS for the treatment of colorectal cancer, including a reduced risk of trocar-related complications, reduced postoperative pain, improved convalescence, and improved cosmetic results [10–14]. However, despite its encouraging results, SILS is technically challenging because of the associated limited instrument movement, loss of triangulation, and in-line viewing, and these technical difficulties may lead to prolonged operation time, increased complications, and uncertain oncological outcomes [12, 14–16]. In addition, in the view of Hamabe et al., the technical difficulties of SILS were more evident in cases of sigmoid and rectal cancer, especially when rectal transection and double-stapling anastomosis were performed intracorporeally through the umbilical incision [16]. To minimize the abdominal trauma and the technical difficulties, the use of single-incision plus one port laparoscopic surgery (SILS plus one), which includes an additional port in the right lower quadrant to SILS for sigmoid and rectal cancer, has gained increasing attention from colorectal surgeons [16, 17]. Adding a port in the right lower quadrant can overcome the technical difficulties and is also convenient for drainage, which makes it possible to effectively evaluate and conservatively manage bleeding, anastomotic leakage, and chyle leakage [16, 17].

Many studies have reported that SILS plus one is feasible for colorectal cancer treatment and offers short-term safety comparable to that of CLS [15, 17]. However, the published studies had several limitations, such as a retrospective design, small samples, and poor methodologically quality [15, 17]. There is still no available evidence from large-scale, randomized controlled trials assessing the feasibility, short-term safety, long-term oncological safety, and potential benefits of SILS compared with CLS for rectosigmoid cancer.

### Objective and hypothesis

The objective of this trial is to evaluate the feasibility, short-term safety, long-term oncological safety, and potential benefits of SILS plus one compared with CLS for the radical resection of rectosigmoid cancer in a randomized controlled trial. The hypothesis is that SILS plus one is feasible for the radical resection of rectosigmoid cancer and offers short-term safety and long-term oncological safety comparable to that of CLS. In addition, SILS plus one provides better cosmetic results and faster convalescence compared with CLS.

## Methods/Design

### Setting

This is a single-center, open-label, noninferiority, randomized controlled trial. The trial is being undertaken at Nanfang Hospital of Southern Medical University, Guangzhou, Guangdong, China, which performs 450 colorectal cancer resections per year. Recruitment began on April 16, 2014, and the trial is expected to proceed for 96 months.

### Participants

Patients aged 18 to 80 years with rectosigmoid cancer diagnosed as cT1-4aN0-2 M0 lesions via abdominal computed tomography (CT) and colonoscopy according to the 7th Edition of the American Joint Committee on Cancer (AJCC) Cancer Staging Manual [18] and a tumor size no larger than 5 cm will be further screened for inclusion (Table 1) by a designated investigator.

**Table 1:** Inclusion, exclusion, and withdrawal criteria

Inclusion criteria	Exclusion criteria	Withdrawal criteria
• 18 years < age < 80 years	• BMI > 30 kg/m^2^	• Invasion of adjacent structures or distant metastasis
• Tumor located in the rectosigmoid (defined as 10 to 30 cm from the anal verge, measured via colonoscopy or EUS)	• Pregnant or lactating women	• Inability to undergo surgery or anesthesia because of a changing illness state
• Pathological rectosigmoid cancer	• Severe mental disease	• Changing illness state requires an emergency operation
• Clinically diagnosed cT1-4aN0-2 M0 lesions according to the 7th Edition of the AJCC Cancer Staging Manual (measured using abdominal CT and colonoscopy or EUS)	• Previous abdominal surgery (except extraperitoneal surgery)	• Intraoperative colon irrigation
• Tumor size of 5 cm or less	• Emergency operation to treat complications (bleeding, perforation, or obstruction) caused by colorectal cancer	• Serious protocol violations
• ECOG performance status of 0 to 1	• Need for simultaneous surgery for another disease	• Patient required to withdraw
• ASA classification I to III	• Malignant disease within the previous 5 years (except superficial squamous or basal cell cancer of the skin or in situ cancer of the cervix)	
• Informed consent	• Nonspeaker of Chinese or English	

*EUS* endoscopic ultrasonography, *AJCC* American Joint Committee on Cancer, *CT* computed tomography, *ECOG* Eastern Cooperative Oncology Group, *ASA* American Society of Anesthesiologists, *BMI* body mass index

### Recruitment and trial timeline

**Fig. 1:**
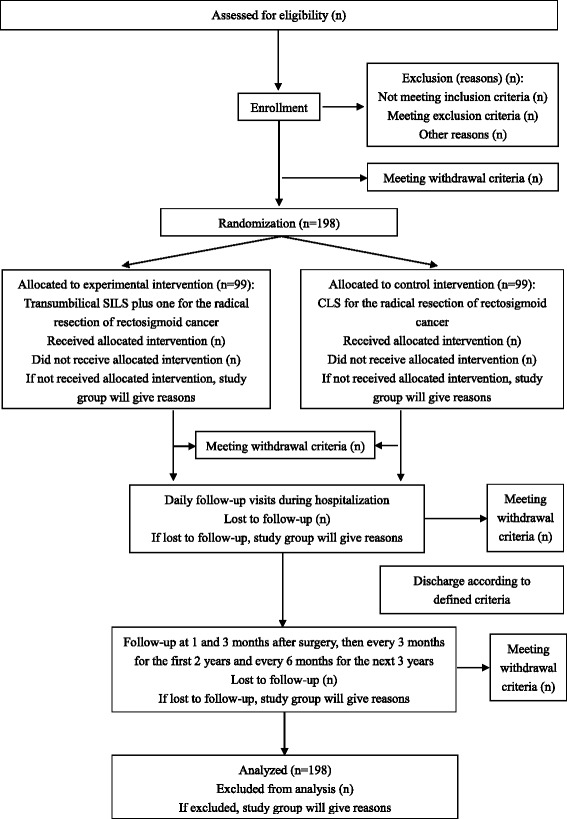
Time schedule and flow of participants

After being screened for inclusion and exclusion criteria, the potential participants will be verbally introduced to the study by an investigator. A signed written informed consent will be obtained from each patient before his or her inclusion in the study. A copy of the signed consent will be given to the participant, and the original consent will be kept in the hospital. Surgery will be performed within 7 days of patient enrollment or, if it is not, the reason will be recorded in the case report form (CRF). After enrollment, the baseline demographic and clinical characteristics of the participant will be measured preoperatively (Table 2), and any participants who meet the withdrawal criteria (Table 1) will be withdrawn from the trial. The flow of participants and time schedule are presented in Fig. 1.

**Table 2:** Preoperative and postoperative outcome parameters and schedule of study visits and follow-up

Measures	Preoperative	Daily in-hospital study visits							Follow-up														
		POD 0^b^	POD 1	POD 2	POD 3	POD 4	POD 5	POD ≥6	M1	M3	M6	M9	M12	M15	M18	M21	M24	M30	M36	M42	M48	M54	M60
Inflammatory parameters (WBC, NE %, CRP, IL-6, TNF-α)	X^a^	X^c^	X^c^			X^c^																	
VAS score			X	X	X		X																
Short-term recovery^d^			X	X	X	X	X	X															
Post-voiding residual urine	X^a^				X																		
EORTC QLQ-C30	X^a^								X	X	X												
EORTC QLQ-CR29	X^a^								X	X	X												
IPSS	X^a^								X	X	X												
BIQ									X	X	X												
Abdominal incision photograph		X							X	X	X												
Physical examination	X^a^								X	X	X	X	X	X	X	X	X	X	X	X	X	X	X
CEA, CA72-4, CA19-9	X									X	X	X	X	X	X	X	X	X	X	X	X	X	X
Chest radiography	X									X	X	X	X	X	X	X	X	X	X	X	X	X	X
Liver ultrasonography										X		X		X		X		X		X		X	
Abdominal and pelvic CT scan	X										X		X		X		X		X		X		X
Colonoscopy or EUS	X										X		X		X		X		X		X		X
Adverse event		X	X	X	X	X	X	X	X	X	X	X	X	X	X	X	X	X	X	X	X	X	X

^a^Preoperative study visits will be scheduled within 7 days before surgery; ^b^The study visit on POD 0 will be scheduled during and after surgery; ^c^Inflammatory parameters will be measured at postoperative 4 hours, 24 hours, and 96 hours after skin suture; ^d^Short-term recovery, including time to first flatus, time to resuming liquid diet and soft diet, time to ambulating independently and discharge. POD, postoperative day; M, month; WBC, white blood cells; NE %, neutrophil percentage; CRP, C-reactive protein; IL-6, interleukin-6; TNF-α, tumor necrosis factor-α; VAS, visual analogue scale; QLQ-C30, Quality of Life Questionnaire-Core 30; QLQ-CR29, Quality of Life Questionnaire-Colorectal 29; IPSS, International Prostatic Symptom Score; BIQ, Body Image Questionnaire; CEA, carcinoembryonic antigen; CA 72-4, carbohydrate antigen 72-4; CA 19-9, carbohydrate antigen 19-9; CT, computed tomography; EUS, endoscopic ultrasonography

From January 2012 to December 2013, 145 patients at Nanfang Hospital met the inclusion criteria. Based on our experiences during our single-incision laparoscopic appendectomy study, we expect a recruitment rate of 90 to 100 % [19]. No provisions or financial compensation will be offered as recruitment strategies. We expect to recruit 198 patients within 36 months, and the trial is designed to proceed for 96 months.

### Randomization, allocation concealment, and blinding

The participants will be randomized at 1:1 ratio to receive either SILS plus one or CLS. A simple random allocation sequence will be computer generated using SPSS version 13.0 (SPSS, Inc., Chicago, IL, USA) by a third party. A nurse will give the surgeon the patient’s random number and group assignment in an identical, opaque, sealed envelope the day before surgery. The operation data will be recorded, and the abdominal incision will be photographed by an investigator who will not be involved further in the trial. An investigator who is not involved with the surgery group will perform the macroscopic examination and photograph the dissected, unfixed specimen. Detailed macroscopic and microscopic examinations of the dissected fixed specimens according to the AJCC guidelines will be performed by a pathologist who is blind to the patient’s treatment assignment [18]. An independent investigator with extensive colorectal experience who is aware of the participant’s assignment will be responsible for assessing early morbidity. An investigator will be in charge of changing the wound dressing but will not be involved further in the trial. This investigator will report any cases of wound complications to the morbidity assessor. The patients’ study-related visits to the hospital will be conducted by an investigator who is blind to participant assignment. All of the investigators will be unblinded at the patient’s discharge. The statistician will be blind to participant assignment.

### Interventions

Surgery will be performed by investigators with ≥ 100 successful laparoscopic colorectal resections and ≥ 10 transumbilical SILS plus one colorectal resections.

#### Experimental intervention

**Fig. 2:**
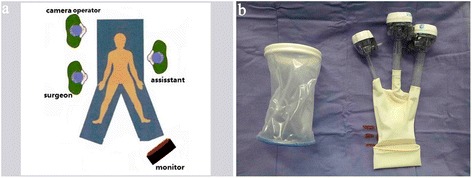
Operative position and single-incision multichannel device. **a** The patient is placed in a lithotomy position. The surgeon stands at the patient’s right side with the first assistant at the left side, while the camera operator stands beside the patient’s right shoulder with the monitor placed beside the patient’s left leg. **b** The homemade multichannel device comprises a soft tissue retractor and a surgical glove

After general anesthesia, the patient will be placed in a lithotomy position on the table. The positions of the surgeon, assistant, and camera operator are depicted in Fig. 2a.

**Fig. 3:**
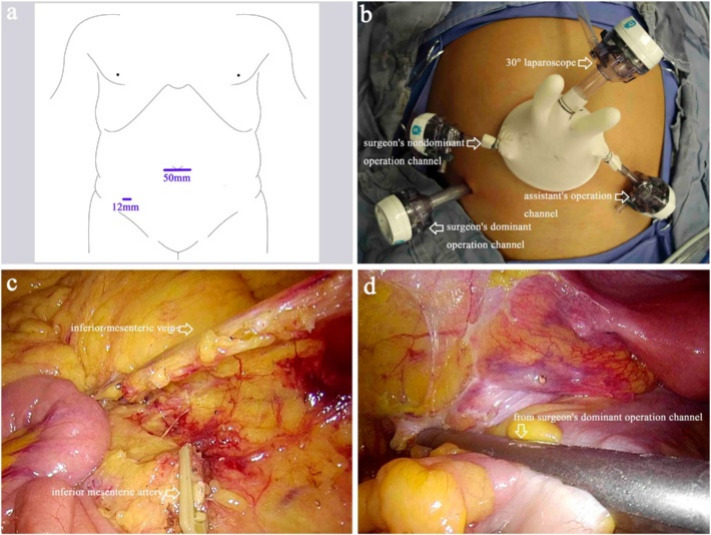
Trocar positions and surgical procedure for the experimental intervention group. **a**, **b** Trocars and instrument positions for single incision plus one port laparoscopic surgery (SILS plus one). **c** Ligation of the inferior mesenteric artery and vein. **d** The distal rectum is dissected by inserting a linear stapling device through the surgeon’s dominant operation channel

An initial 5-cm periumbilical transverse incision will be made. Then, the homemade multichannel device, comprising a soft tissue retractor and a surgical glove, will be placed at the umbilical incision (Fig. 2b). Two 12-mm trocars and one 5-mm trocar will be inserted through the glove fingers as the observation port for a 10-mm 30° laparoscope, the surgeon’s nondominant operation channel, and the assistant’s operation channel, respectively. A pneumoperitoneum of 12-13 mmHg will be established and maintained. One 12-mm trocar will be placed in the right lower quadrant under laparoscopic view as the surgeon’s dominant operation channel (Fig. 3a,b).

After the abdomen is explored with the laparoscope, the patient will be placed in the Trendelenburg position with the left side elevated 20 to 30°. High ligation of the inferior mesenteric artery approximately 4 cm above the abdominal aortic bifurcation will be performed with a clip for D3 lymphadenectomy (Fig. 3c). Subsequently, the inferior mesenteric vein will be ligated near the duodenojejunal peritoneal fold (Fig. 3c). Then, the medial-to-lateral dissection will be performed. The distal rectum will be dissected by inserting a linear stapling device through the surgeon’s dominant operation channel (Fig. 3d). The surgical specimens will be removed through the umbilical incision under the soft tissue retractor and dissected extracorporeally. Intracorporeal end-to-end anastomosis will be performed using a circular stapler inserted through the anus and tested with air insufflation. A drainage tube will be placed in the pelvic cavity through the surgeon’s dominant operation channel. The fascia and the skin will be reapproximated with simple interrupted absorbable and nonabsorbable sutures, respectively. Then, the incision length will be measured, and the wound will be dressed. Additional movie files show the operation procedure in more detail (see Additional file 1).

#### Control intervention

After general anesthesia, the position of the patient, the surgeon, the assistant and the camera operator will be the same as described for the experimental group (Fig. 2a).

**Fig. 4:**
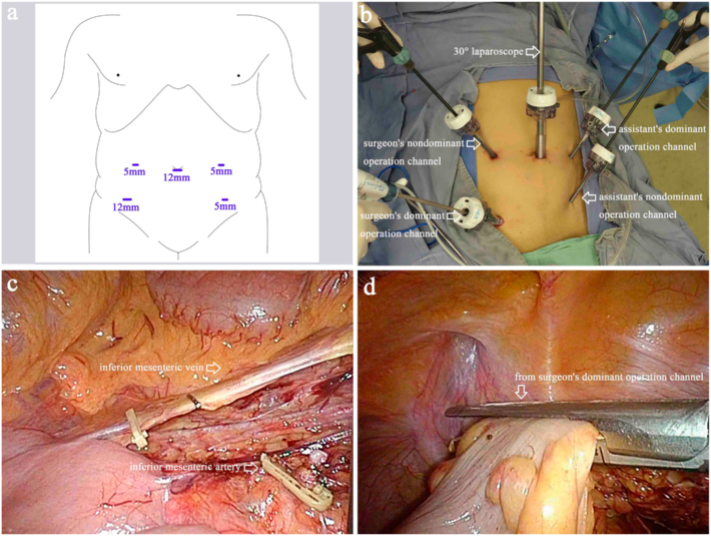
Trocar positions and surgical procedure for the control intervention group. **a**, **b** Trocars and instrument positions for conventional laparoscopic surgery (CLS). **c** Ligation of the inferior mesenteric artery and vein. **d** The distal rectum is dissected by inserting a linear stapling device through the surgeon’s dominant operation channel

A periumbilical incision for a 12-mm trocar will be made as the observation port for a 10-mm 30° laparoscope. After a 12- to 13-mmHg carbon dioxide pneumoperitoneum is created, additional transabdominal trocars will be placed under laparoscopic view: one 12-mm trocar in the right lower quadrant as the surgeon’s dominant operation channel, one 5-mm trocar in the left quadrant as the assistant’s nondominant operation channel, and two 5-mm trocars in the right and left upper quadrants as the surgeon’s nondominant and the assistant’s dominant operation channel, respectively (Fig. 4a,b).

**Fig. 5:**
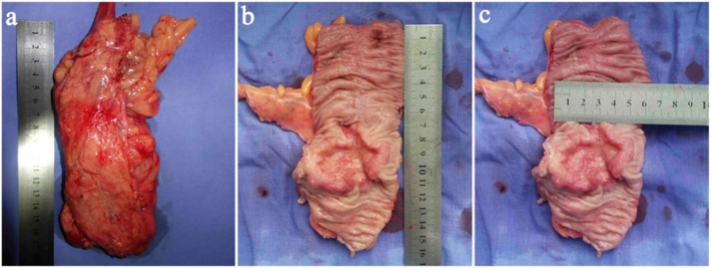
Specimen measurement. **a** Macroscopic quality of the complete mesocolic excision. **b** Length of the proximal and distal dissection margins. **c** Tumor diameter

After laparoscopic exploration of the abdomen, the surgical procedure will be performed in the same manner described for the experimental group (Fig. 4c,d). After distal rectal colon dissection, an extension incision will be performed, and the rectosigmoid colon will be extracted under a soft tissue retractor and dissected extracorporeally. The procedure that follows will be the same as that described for the experimental group:

1. Mobilization of the splenic flexure will not be performed routinely except in cases of a lack of redundancy of the sigmoid colon [14, 20].
2. In the CLS group, the length and the location of the extension incision will be determined by the surgeon.
3. Adding ports or converting to open surgery will be allowed at the surgeon’s discretion if technical difficulties, the patient’s safety, simultaneous surgery, or unexpected conditions requiring conversion.
4. Only standard straight laparoscopic instruments will be used for the operation.
5. A temporary protective loop ileostomy will be allowed for high-risk patients, such as those taking steroid medication, those with an abscess discovered during surgery, or those with a poor nutritional state [21], at the surgeon’s discretion.
6. The operation procedures for both intervention groups will be digitally recorded and left unedited.
7. Immediately after the operation, an investigator will check the macroscopic quality of the complete mesocolic excision [22] (Fig. 5a) and measure the length of the proximal and distal dissection margins (Fig. 5b) and the tumor diameter (Fig. 5c).

### Perioperative management, discharge, and follow-up

Nutritional support will be performed according to Nutritional Risk Screening 2002 [23] and ERAS Society recommendations [24]. Polyethylene glycol electrolyte solution (2.5 L) will be administered the day before surgery for bowel preparation. A single short dose of prophylactic antibiotics (second-generation cephalosporins) will be given intravenously 30 minutes before surgery [24]. Systemic prophylactic antibiotics (second-generation cephalosporins) will also be given intravenously within 72 hours postoperatively. Nasogastric tubes will not be routinely used [24]. Postoperative patient-controlled opioid-based intravenous analgesia will be routinely administered directly after surgery in the recovery room and discontinued on postoperative day (POD) 2 [25]. Additional analgesics will be allowed in cases of breakthrough pain as recommended by the World Health Organization Analgesic Ladder and at the discretion of the treating ward physician [26]. The patient’s urinary catheter will be removed on POD 3. After the first spontaneous voiding, a bladder ultrasound will be performed to measure the post-voiding residual urine. If the patient has symptoms of urinary retention or is unable to pass urine spontaneously 6 hours after catheter removal, a catheter will be reinserted for at least 24 hours [27]. The drain will be removed at the surgeon’s discretion and based on the amount of drainage and the properties of the drained fluid. Participants will be discharged once they are able to tolerate a soft diet and ambulate independently (defined as being able to walk a predefined distance or go to the bathroom independently [28]).

Postoperative adjuvant chemotherapy will be performed according to the NCCN guideline [29, 30] at the surgeon’s discretion and if the participant is willing. Each participant will be followed up at 1 and 3 months after surgery, then every 3 months for the first 2 years and every 6 months for the next 3 years. Quality of life will be measured with the European Organization for Research and Treatment of Cancer (EORTC) Quality of Life Questionnaire-Core 30 (QLQ-C30) [31], the EORTC Quality of Life Questionnaire-Colorectal 29(QLQ-CR29) [32], and the International Prostate Symptom Score (IPSS) [33] preoperatively and 1 month, 3 months, and 6 months postoperatively. The participants will be evaluated for tumor recurrence using the following methods: physical examination, carcinoembryonic antigen (CEA), carbohydrate antigen 72-4 (CA 72-4), and carbohydrate antigen 19-9 (CA 19-9). Chest radiographs will be obtained every three months for the first 2 years and then every 6 months for the next 3 years. Liver ultrasonography will be performed for the first time at the third postoperative month, then every 6 months for the first 2 years and annually for the next 3 years. Colonoscopy or EUS and abdominal and pelvic CT scans will be performed every 6 months for the first 2 years and then annually for the next 3 years. If the participant fails to return for follow-up visits, he or she will be contacted by phone or email to complete the follow-up data.

### Risk-benefit ratio

The potential benefits of SILS compared with CLS are expected to be fewer trocar-related complications, reduced postoperative pain, faster convalescence, and improved cosmetic results [10–14]. The potential risks of SILS compared with CLS for colorectal cancer treatment are prolonged operation time, increased complications, and uncertain oncological outcomes. However, several retrospective studies have reported that the operation time, complications, pathologic outcomes, and 3- to 5-year DFS and OS for SILS were comparable to those of CLS for colorectal cancer [1, 14, 34, 35].

### Outcome measures

#### Primary endpoints

The primary outcome is early morbidity, which will be divided into morbidity observed during operation and morbidity observed within 30 days after surgery. Early morbidity will be assessed on POD 30 and classified according to the Clavien-Dindo classification [36].

#### Secondary endpoints

Secondary endpoints will be measured intraoperatively, at daily study visits, and at 1, 3, 6, 36, and 60 months postoperatively. Operative outcomes include operation time, estimated blood loss during operation, and incision length. Pathological outcomes compromise tumor size, length of proximal and distal resection margins, and number of harvested lymph nodes and will be determined postoperatively by pathological examination. Postoperative inflammation and immune response (white blood cells [WBC], neutrophil percentage [NE %], C-reactive protein [CRP], interleukin-6 [IL-6], and tumor necrosis factor-α [TNF-α]) will be measured at 4 hours, 24 hours, and 96 hours postoperatively. Postoperative recovery (time to first ambulation, flatus, liquid diet, soft diet and duration of hospital stay) will be measured daily in-hospital and determined using participant self-report. Pain intensity will be measured using a horizontal visual analogue scale (VAS; a 0 to 10 cm scale with 0 at the left edge representing no wound pain, and 10 at the right edge representing wound pain as bad as it could possibly be) [37] at rest on POD 1, 2, 3, and 5. Additional postoperative analgesic use will be recorded in the CRF. Body image and cosmesis will be measured using the Body Image Questionnaire (BIQ) [38] at 1, 3, and 6 months postoperatively. The 3-year disease-free survival (DFS) and 5-year overall survival (OS) will be measured at 36 and 60 months postoperatively. Confirmation of recurrence will require either the detection of radiologically apparent lesions that increase in size over time or a pathology evaluation. The DFS will be calculated from the time of randomization to the time of either local or distant progression or death (from any cause and in the absence of evidence of recurrence) in participants who have undergone curative surgery. The OS will be calculated from the time of randomization to the time of death from any cause. All measurements and time points are listed in Table 2.

### Data management

Data will be recorded in the CRF by an investigator and reviewed by another investigator for completeness before it is transferred into the trial database. Any missing data will be identified and drawn from source data or from the participants. The CRF will then be transferred into the trial database and double-checked to ensure accurate transfer. Each participant will be given an allocation number according to their randomization. To maintain participant confidentiality, all data will be identified by allocation number, and only the investigators will be able to link the allocation number to the participant’s identification using a list saved separately from the database. All study-related participant information will be stored in a specified investigator’s office in locked filing cabinets. The trial database, photographs and videos will be double saved. In case of withdrawal, follow-up and data collection will continue with the participant’s permission. An investigator will review the database to ensure accurate data collection using descriptive statistics to check for missing data and out-of range values. Any unclear data will be traced to the original medical records. Upon the completion of the trial, all study-related data and trial documents will be archived securely and retained for a minimum of 10 years at Nanfang Hospital.

### Safety and reporting of serious adverse events

All adverse events (AEs, defined according to the Common Terminology Criteria for Adverse Events (CTCAE) version 4.0 [39] and the Accordion Severity Grading System (TASGS) [40]), will be evaluated for severity and causality. Severity will be graded according to the CTCAE [39] and TASGS [40]. Causality will be graded as none, unlikely, possibly, probably, and definitely related to the intervention. Expectedness will be used to evaluate the AEs. Any serious adverse events (SAEs, defined according to the International Conference on Harmonisation (ICH) GCP E6 guidelines [41], TASGS [40], and CTCAE [39]) will be reported to the chief investigator (CI) within 24 h of being noted. If the CI considers an SAE unexpected and related to the study intervention, he will report it to the Medical Ethics Committee (MEC) within 72 h of being notified, and a detailed medical report will be needed within 15 days. The study’s steering committee, which is organized by the authors of the protocol and the Department of General Surgery, Nanfang Hospital, will be responsible for overseeing the study’s progress and safety and will meet to evaluate morbidity and AEs at least three times: after the randomization of 25 %, 50 %, and 75 % of the participants. If the number of treatments related to SAE occurrence is more than 3 % (n = 2) of the sample size in each group, patient enrollment will be terminated immediately, and the MEC will reassess the safety of the trial.

### Statistical methods

#### Sample size

The sample size was determined for the primary endpoint: early morbidity measured on POD 30. Based on our database, the early morbidity rate of CLS for colorectal cancer is 15.2 %; for SILS plus one for colorectal cancer, the early morbidity rate is 11.1 %. The sample size was determined according to the following:

1. Hypothesis: the early morbidity of SILS plus one is not higher than that of CLS (noninferiority analysis).
2. We set the noninferiority margin at 10 %.
3. A one-sided analysis with a type I error (α) of 2.5 % and a power (1-β) of 0.80 was assumed.

Based on these assumptions, a sample size of 90 participants per arm was calculated using the NCSS-PASS (11^th^ edition, NCSS, LLC, Utah, USA). Assuming a drop-out rate of 10 %, the total number of participants needed per arm is 99, resulting in a total of 198 participants for the primary aim.

#### Statistical analysis

The statistical analysis will be performed using SPSS version 13.0 (SPSS, Inc., Chicago, IL, USA). A two-sided p < 0.05 will be considered significant. Descriptive statistics will be used for baseline characteristics. For categorical variables, including the primary outcome, a χ^2^ test or Fisher’s exact test will be applied. For continuous variables, Student’s t test or the Mann-Whitney U-test will be applied. Kaplan-Meier curves and the log-rank test will be used to analyze the OS and DFS differences between the two groups. We do not plan to perform interim analyses. Missing values will not be imputed. Any outcomes of interest analysis will be performed based on the intention-to-treat population and per-protocol population (PPP), and a standard sensitivity analysis will be performed based on the PPP.

### Ethical considerations

The study will be performed according to the Declaration of Helsinki [42] and the ICH GCP E6 guidelines [41]. The Medical Ethics Committee of Nanfang Hospital reviewed and approved this study on March 19, 2014 (reference number: NFEC-2014-026). The Medical Ethics Committee of Nanfang Hospital will be informed of significant protocol amendments.

Only the patients who signed the informed consent will be included in the study. The participants will be informed that the participation in the trial is voluntary and that they can withdraw consent at any time without giving reasons.

### Dissemination policy

This trial is intended for publication in international peer-reviewed journals. The results of this study will also be presented at internationally relevant scientific meetings. The progress and the results of the study will be saved at Clinicaltrials.gov to allow general access to documented findings.

### Registration

This trial was registered with ClinicalTrials.gov (https://www.clinicaltrials.gov/ct2/show/NCT02117557?term=single&cond=colon+cancer&cntry1=ES%3ACN&rank=11) under the registration number NCT02117557 on April 16, 2014.

### Protocol version

This manuscript refers to the second version of the full study protocol issued on 20 December 2013. Protocol modifications will be reported to all investigators, the MEC, all trial participants, and the journal.

## Discussion

Although the SILS plus one technique has been adopted in clinical use because it provides most of the benefits of SILS and CLS and minimizes the risks associated with SILS [15, 17], as far as we know, there are only two large-scale randomized controlled trials with more than 100 samples comparing SILS with CLS for colorectal cancer treatment. One is the SILVERMAN1 trial for right colonic cancer [43], and the other is the Multicenter Single-Port Colectomy trial for colon adenocarcinoma excluding distal transverse colon cancer, splenic flexure colon cancer, and descending colon cancer [44]. There are no published results. To the best of our knowledge, no large-scale RCT has focused on the risks and benefits of SILS plus one compared with CLS for rectosigmoid cancer treatment.

Early morbidity was chosen as a primary endpoint of this trial because early morbidity is an indication of short-term safety, which is a prerequisite for the general clinical application of a new surgical technique [14]. Moreover, early morbidity is a measure of surgical recovery for patients undergoing major abdominal surgery [28]. Among all types of early morbidities, we will focus on intra-abdominal morbidity and trocar-related morbidity, including intraoperative morbidity (such as injuries and bleeding during surgery) and 30-day postoperative morbidity (such as anastomotic leakage, anastomotic bleeding, intro-abdominal bleeding, incision complications and trocar-related complications). If the morbidity differs between SILS plus and CLS for rectosigmoid cancer, the technical difficulties (such as limited instrument movement, loss of triangulation, difficulty with rectal transection and intracorporeal double-stapling of anastomoses) and the different trocar numbers may be the main reasons. Moreover, the operation time and estimated blood loss during surgery have also been used to evaluate the safety of surgical techniques.

Oncological safety is an important measurement for a new surgical technique in the field of radical cancer resection, and the most powerful evidence of oncological safety is the long-term DFS and OS. In 2014, a case-match study compared 27 SILS patients with 27 CLS patients treated for colorectal cancer, and the 5-year local recurrence rate was 3.7 % versus 0 (p = 0.55), the distant metastasis rate was 14.8 % versus 3.7 % (p = 0.15), and the OS was 92.6 % versus 96.3 % (p = 0.66) for SILS and CLS, respectively [35]. In the same year, in a propensity-score matching analysis, Kim et al. compared SILS (n = 60) with CLS (n = 120) for sigmoid cancer. The 3-year DFS was 89.5 % and 87.4 % (p = 0.751), and the OS was 94.5 % and 97.1 % (p = 0.223) for SILS and CLS, respectively [14]. As the cases and follow-ups accumulated, Yun et al. recently reported that SILS (n = 239) colorectomy showed an equivalent of 48 months DFS (89.8 % versus 89.9 %, p = 0.548) and OS (98.7 % versus 98.6 %, p = 0.971) compared with CLS (n = 239) for colon cancer [1]. Although these three studies suggest that the oncological safety of SILS is comparable to that of CLS for colorectal cancer, the retrospective design, small samples and poor methodological quality of these studies might have resulted in selection bias and evaluation bias, which would largely reduce the believability of the study results. Considering all of the above reasons, it is necessary to evaluate the oncological safety of SILS plus one for rectosigmoid cancer using the 3-year DFS and the 5-year OS. Evaluating the long-term DFS and OS of patients with colorectal cancer requires long-term follow-up; consequently, the assessment of the pathological outcomes (such as the tumor size, length of the proximal and distal resection margins, and the number of harvested lymph nodes removed) of each specimen is essential for predicting oncological safety.

As an essential principle of enhanced recovery, minimally invasive surgery has been championed by a growing number of surgeons [24]. It seems obvious that the SILS plus one technique will enhance recovery compared with CLS by reducing abdominal trauma; however, as the evidence accumulated, there were arguments over whether the reduction of trocar incision is enough to enhance patients’ recovery [1, 13, 14, 34, 45]. To settle the arguments, we will use pain intensity, duration of hospital stay, time to first ambulation, bowel function (time to first flatus), eating habits (time to resuming liquid diet and soft diet) and inflammatory and immune responses (WBC, NE %, CRP, IL-6 and TNF-α) to evaluate patients’ recovery after abdominal surgery [28]. Among the above-mentioned measurements, pain is the most commonly reported symptom and the most subjective one [28]. To transform subjective pain into an objective score, we will use the VAS scale, which is the most sensitive pain scale; compared with the Numerical Rating Scale and the Four-point Verbal Rating Scale, it can discriminate between small changes in pain intensity [46]. We decided to measure patient pain intensity at rest because doing so may eliminate the effect of different levels of mobility on pain intensity. Additionally, we will record the use of additional postoperative analgesics for breakthrough pain, which will influence pain intensity, for further analysis.

The cosmetic advantage of SILS plus one over CLS seems obvious, and it seems to be a permanent advantage of SILS plus one. However, at present, no study available assesses whether reducing the use of abdominal trocars will affect body image, cosmesis, and patient self-confidence. We will use the BIQ to evaluate the patient’s subjective feelings about the cosmesis, and we will use photographs of the incision and the length of incision to supplement the cosmesis evaluation [38].

This trial has potential limitations. 1) The trial will take place in only one center, which may impact participant recruitment or limit the generalizability of the results to other hospitals. 2) The surgeons, participants, and investigators who record the surgical information, photograph the incisions, change the wound dressings, and perform the study visit after each participant’s discharge will be aware of the group assignment, which may cause biased estimates of the treatment effect. However, because of the special nature of nonpharmacologic trials, it is impossible to blind the surgeon [47]. Although in this trial, the surgeons, the investigators mentioned above, and the participants are not blinded, the pathologists, statistician, and investigators who evaluate the unfixed specimens and perform the study visits during the patients’ hospital stays will be blinded to the group assignment. Moreover, the participants, surgeons, and investigators will not be allowed to reveal the group assignments to the others. These rules can reduce the treatment effect estimation bias.

Despite its increasing clinical application, the risks and benefits of SILS plus one for rectosigmoid cancer remain unclear. This trial is the first study to compare SILS plus one with CLS for the radical resection of rectosigmoid cancer in a large-scale, randomized, controlled trial setting. The results of this trial will provide valuable clinical evidence for the objective assessment of the feasibility, short-term safety, long-term oncological safety, and potential benefits of SILS plus one compared with CLS for the radical resection of rectosigmoid cancer.

### Trial status

The trial recruitment started in April 2014. As of June 18, 2015, 82 patients have been registered and randomized.

## Additional file

Additional file 1:
**Operation procedure 1-6.** (ZIP 89284 kb)

## Abbreviations

AEs: adverse events
AJCC: American Joint Committee on Cancer
BIQ: Body Image Questionnaire
CA 72-4: carbohydrate antigen 72-4
CA 19-9: carbohydrate antigen 19-9
CEA: carcinoembryonic antigen
CI: chief investigator
CLS: conventional laparoscopic surgery
CRF: case report form
CRP: C-reactive protein
CT: computed tomography
CTCAE: Common Terminology Criteria for Adverse Events
DFS: disease-free survival
EORTC: European Organization for Research and Treatment of Cancer
ERAS: Enhanced Recovery After Surgery
EUS: endoscopic ultrasonography
ICH: International Conference on Harmonisation
IL-6: interleukin-6
IPSS: International Prostatic Symptom Score
MEC: Medical Ethics Committee
NE %: neutrophil percentage
OS: overall survival
POD: postoperative day
PPP: per-protocol population
QLQ-C30: Quality of Life Questionnaire-Core 30
QLQ-CR29: Quality of Life Questionnaire-Colorectal 29
SAEs: serious adverse events
SILS: single-incision laparoscopic surgery
SILS plus one: single-incision plus one port laparoscopic surgery
TASGS: The Accordion Severity Grading System
TNF-α: tumor necrosis factor-α
VAS: visual analogue scale
WBC: white blood cells
